# Thermo-voltage measurements of atomic contacts at low temperature

**DOI:** 10.3762/bjnano.7.68

**Published:** 2016-05-30

**Authors:** Ayelet Ofarim, Bastian Kopp, Thomas Möller, León Martin, Johannes Boneberg, Paul Leiderer, Elke Scheer

**Affiliations:** 1 Department of Physics, University of Konstanz, Universitätsstraße 10, 78464 Konstanz, Germany

**Keywords:** atomic contacts, finite element simulations, laser heating, low temperature, mechanically controllable break-junction, temperature determination, thermopower

## Abstract

We report the development of a novel method to determine the thermopower of atomic-sized gold contacts at low temperature. For these measurements a mechanically controllable break junction (MCBJ) system is used and a laser source generates a temperature difference of a few kelvins across the junction to create a thermo-voltage. Since the temperature difference enters directly into the Seebeck coefficient *S* = −Δ*V*/Δ*T*, the determination of the temperature plays an important role. We present a method for the determination of the temperature difference using a combination of a finite element simulation, which reveals the temperature distribution of the sample, and the measurement of the resistance change due to laser heating of sensor leads on both sides next to the junction. Our results for the measured thermopower are in agreement with recent reports in the literature.

## Introduction

The energy and heat management in electronic devices has become a challenge in recent years due to the down-scaling of electronic components to the nanoscale, where the transport is governed by quantum-mechanical properties, which are partially not explored thoroughly yet. This includes solid-state semiconducting devices [1] and organic semiconductors, ultrathin metal wires or single-molecule junctions. In particular, the thermopower has become a property of utmost interest because it is decisive for the conversion of temperature gradients into electrical energy and for the local energy dissipation. More fundamentally, the study of thermoelectric effects in atomic-scale nanostructures and in molecular junctions gives important additional information of charge transport [2–4]. The thermopower is quantified by the Seebeck coefficient *S* = −Δ*V*/Δ*T*, where Δ*V* is the thermo-voltage and Δ*T* is the temperature difference. In general *S* is a function of energy and temperature:

[1]
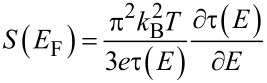


Here *E*_F_ is the Fermi energy, τ(*E*) is the transmission function, *e* is the electron charge, *k*_B_ is the Boltzmann constant and *T* the temperature of the system [5–6]. *S* can adopt both signs and typical values for single-atom and single-molecule contacts range from nV per K to several µV per K.

So far several approaches for thermo-voltage measurements of atomic-scale devices have been performed successfully [7–17]. Experimental techniques such as scanning tunneling microscopy (STM) and the mechanically controlled break-junction (MCBJ) technique allow investigation of transport properties of atomic-scale devices [18]. Therefore, most approaches for measurements of thermo-voltage, or simultaneous measurements of conductance and thermo-voltage, were using STM or MCBJs techniques. When using the STM method, results are given in a statistical manner, mostly around room temperature where the junctions are short-lived and the measurements have to be performed in a transient state [7–8,10]. Therefore correlating the thermopower results to the electrical conductance is not possible for the individual junctions. In most STM realizations, a temperature gradient is achieved by heating the substrate, whereas the tip remains at room temperature, or vice versa. The temperature difference is then assumed to be the difference of the temperature applied to the substrate and the temperature of the tip or is deferred from simulations. In the recent work by Evangeli et al. [10] simultaneous measurements of the conductance and thermo-voltage of metallic point-contacts were performed using STM. In that work pronounced deviations from the behavior of bulk metals were found. The bulk values were retrieved for contacts larger than 10000 atoms in cross section only. Dell et al. [11] studied the charge transport characteristics of a series of organic molecules. Through thermo-voltage measurements, they showed that the transport changes from hole to electron transport, when the number of thiophene dioxide monomers in the molecular backbone increases. A very elegant method for simultaneous measurements of the conductance and the thermopower has recently been presented by Kim et al. who create an oscillating temperature difference across an electromigrated Au–molecule–Au junction by AC Joule heating of a nanopatterned resistor and recording with lock-in technique the local temperature by a scanning probe [17]. However, the conductance of these contacts cannot be tuned in situ.

A MCBJ setup, in contrast, is stable enough to study an individual contact thoroughly, i.e., performing simultaneous measurements of conductance and thermopower. In MCBJ, due to the fact that both electrodes are mechanically and thermally anchored to the same substrate, achieving a variable heat gradient across the junction is much more complex. One of the pioneering measurements of the conductance and thermo-voltage simultaneously were performed in a MCBJ, at low temperature, where the Joule heating of a discrete resistor element at one side of the junction created a temperature gradient and two other resistors, on each side of the junction, were used as thermometers [12]. The resistors were placed at a distance of roughly a millimeter from each other, in the leads of a MCBJ made from a notched gold wire. Temperature differences in the order of several kelvins were achieved at a base temperature of 12 K. Kaneko et al. [16] have also used a notched-wire MCBJ for measuring simultaneously thermo-voltage and conductance at low temperatures. In this case, a heater sheet was placed under the metal leads to create a temperature gradient, and a resistive thermometer was placed on top of each electrode. They observed a negative correlation between the electrical conductance and the thermo-voltage of a benzenedithiol (BDT) junction.

However, this approach cannot straightforwardly be applied to thin-film MCBJ devices where the freestanding length amounts to a few microns only. In this case the temperature gradient is usually achieved by placing a lithographically defined micro-resistor close to one of the leads. Recently, several studies by Tsutsui et al. [13–15] showed simultaneous thermo-voltage and conductance measurements with thin-film MCBJ at room temperature, when the heat input was realized using Joule heating. They showed that the thermo-electric transport properties of BDT single-molecule junctions are extremely sensitive to the configuration between the two metal leads. The temperature difference across the junction was not measured but calculated using a simulated temperature profile.

We have shown recently [19] that a thermo-voltage of few µV can be achieved experimentally in Au–Ag junctions, in which a Ag crystallite bridged a ≈ 500 nm gap between two Au leads at room temperature. In this case, a laser was used as a heat source. The determination of Δ*T* was made through a measurement of the current-dependent thermo-voltage signal where the highest Δ*T* was tens of kelvins. In addition, the possibility to scan the laser beam across the sample was favorable for disentangling thermo-voltage effects from artifacts due to the temperature dependence of the lead resistances and to maximize the effective Δ*T*. The possibility to use the temperature dependence of the lead resistance for the determination of Δ*T* was also applied in [20]. A temperature gradient achieved by a laser source has been used before for contacts realized by pores through a freestanding membrane, but the Δ*T* achieved was only about 50 mK for a relatively wide tunnel junction. Thus the Δ*V* values were at the resolution limit [21]. Moreover, Δ*T* was not measured, but simulations were applied to estimate it.

Here we suggest a new method for the measurement of the temperature gradient across an atomic-scale device. In addition, we compare the measured values with simulations. Our thin-film MCBJ setup enables us to measure thermo-voltage and conductance at low temperature simultaneously in one circuit (“main circuit”) and determine the temperature difference across the junction in the second (“temperature circuit”). At variance to most other approaches, the temperature gradient is achieved by illuminating the sample locally with a focused laser beam.

## Results and Discussion

### Realization of the thermopower measurement

The sample was prepared by electron-beam lithography as described earlier [22] but using Kapton Cirlex as substrate material instead of the typical break junction substrates bronze or stainless steel [18,23–24]. Kapton Cirlex is an electrically insulating and optically opaque material based on polyimide. We use a thickness of 500 µm chosen such that it is mechanically robust yet giving flexibility across a wide temperature range down to 4 K and is thus suitable for low temperature measurements. Apart from being flexible its thermal conductivity is one to two orders of magnitude lower at 4 K than the one of the usual metallic substrates. This supports the creation (and the preservation) of a temperature gradient for thermopower measurements. Due to r.m.s. surface roughness of 1 µm it was necessary to polish the substrate before spin-coating a thin layer (2–3 µm) of polyimide which enhances the planarization of the substrate. Furthermore, the layer serves as a sacrificial layer to be etched in order to create a freestanding Au bridge of approximately 2 µm length. A drawback of using this (thermally and) electronically insulating substrate was the necessity to overcome the charge-caused deterioration of the electron-beam during the lithography process. To do so, substrates were covered by a thin 10 nm Al film on top of the standard photoresist (MAA/PMMA). This metal layer was removed after electron-beam lithography by soaking the sample in a sodium hydroxide solution for few tens of seconds.

**Figure 1:**
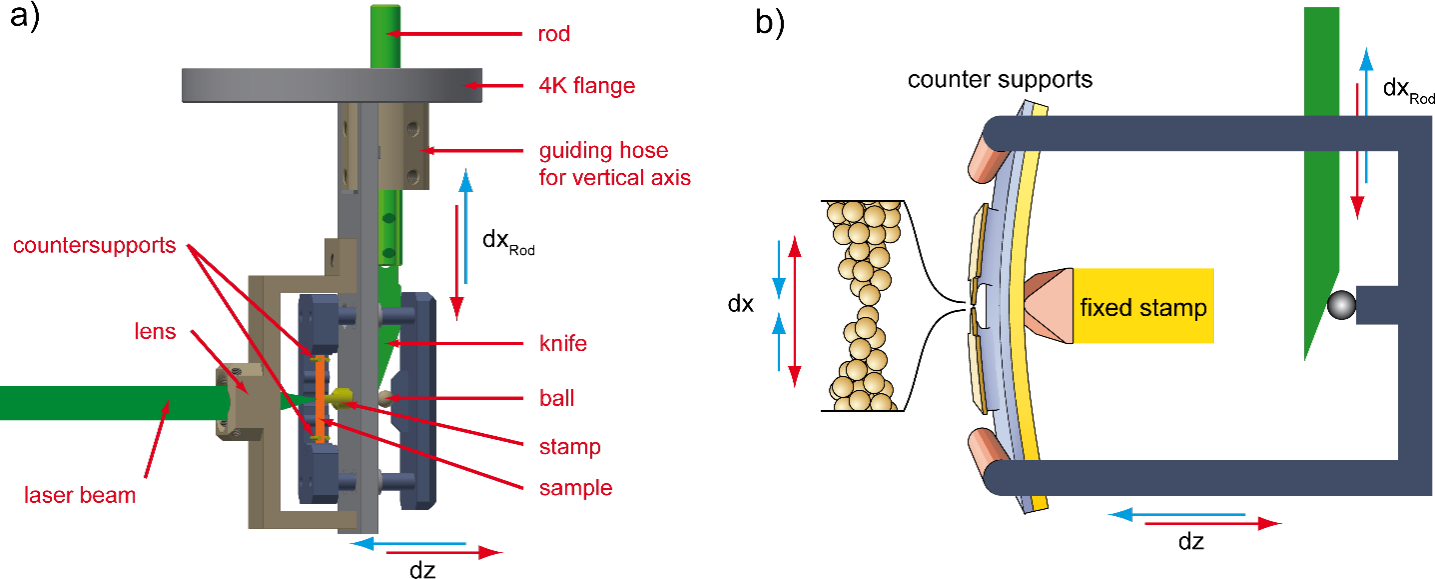
a) Schematics of the MCBJ mechanism. The sample is represented as the orange line and the junction is located in its center. b) Schematics of the movement scheme for clarification. The green-labeled parts indicate the vertical and the blue ones the horizontal movement.

An optical cryostat was used, allowing measurements at 77 K or 4 K. Preliminary measurements have shown the necessity to work at low temperature to enhance temporal stability of the junctions and provide clean conditions owing to cryogenic vacuum. For arranging the atomic-size junctions we designed a MCBJ mechanism that keeps the position of the atomic bridge almost constant while bending the substrate. In addition, the sample is mounted vertically to facilitate optical focusing and scanning. A scheme of the set-up is shown in Figure 1. By turning the rod by a motor we elongate and finally break the constriction of the MCBJ. The rotation of the motor rod is first translated into a vertical movement (d*x*_Rod_) followed by a translation of this movement into a horizontal one (d*z*), by using a knife that pushes a ball. The sample is then bent, so that the horizontal movement is again translated into a now pico-metric distance change of the electrodes to form and break the bridge (d*x*).

**Figure 2:**
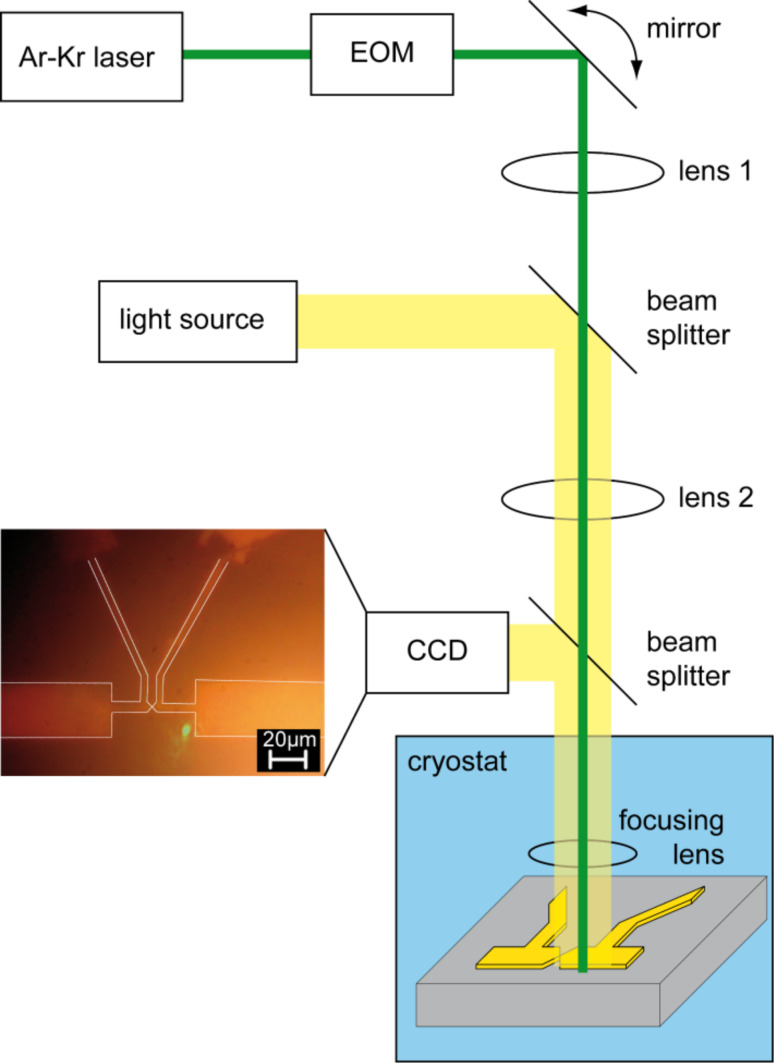
Optical setup: An Ar-Kr laser serves as cw laser source at a wavelength of λ = 514 nm and the beam was chopped by an electro-optical modulator (EOM). Distances between mirror, lenses 1 and 2 and the sample were chosen such that the position of the laser focus on the sample could be adjusted by tilting the mirror. A light source illuminated and a CCD camera monitored the sample. The resulting optical image is shown in the lower left, where the white lines indicate the contour of the Au structure and the laser spot (green point) shows the illuminated position.

To enable irradiation with light of varying pulse lengths, all components of the optical set-up are mounted outside the cryostat except for the last focusing lens, see Figure 2. In the experiments described in this paper we used a continuous-wave Argon Krypton (Ar-Kr) laser at a wavelength of λ = 514 nm. The laser beam was chopped by an electro-optical modulator and was then focused with a variable intensity up to 3 mW onto the sample. The influence of thermal gradients onto atomic-size contacts usually causes geometry changes of the tips due to thermal expansion caused by the deposited energy. For our experiment we obtain from simulations that the distance change between the tips forming the atomic contact is smaller than 1 pm, thereby limiting possible changes of the conductance to less than 10^−2^*G*_0_ [24–25]. In addition, the irradiation with laser light may cause excitations in the Au layer that influence the electronic properties [23–24]. To avoid such unwanted other sources of voltage signals we do not irradiate the metal leads but the substrate nearby, see Figure 2 and choose to focus the laser heating several microns away from the constriction. Distances between mirror, lenses 1 (*f*_1_ = 50 mm) and 2 (*f*_2_ = 150 mm) and the sample were chosen such that the position of the laser focus on the sample could be adjusted by tilting the mirror. The short distance between the objective lens and the sample surface of *f*_3_ = 12.6 mm enables a smallest focus diameter (full-width at half-maximum (FWHM)) of about 12.7 µm (see Methods section). The position of the laser focus on the substrate is monitored by a CCD camera.

**Figure 3:**
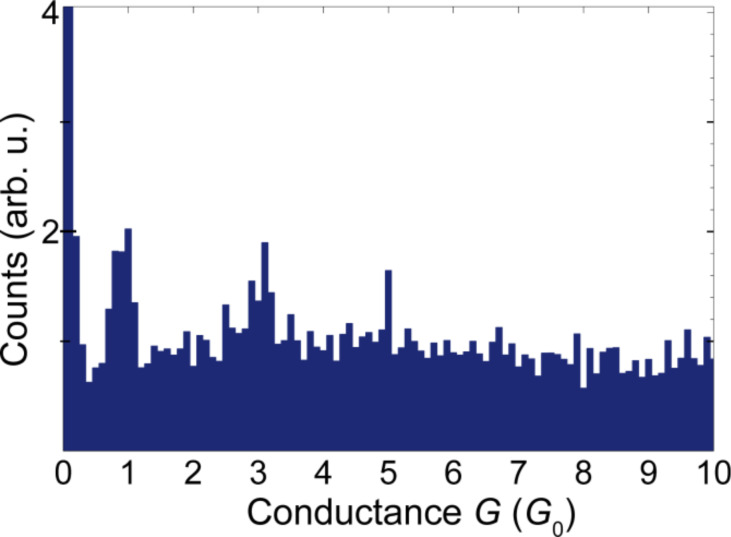
Conductance histogram at 77 K of 220 opening and closing curves recorded with an applied voltage of 5 mV. *G*_0_ = 2*e*^2^/*h* is the conductance quantum.

The sample is controllably broken and closed at low temperature in cryogenic vacuum by bending and flattening the substrate without laser irradiation, while recording the current at an applied bias of 5 mV. Motor control and data collection is performed using a custom-made data acquisition program. This measurement is repeated hundreds of cycles to achieve a large statistical ensemble for the determination of the correlation between the thermo-voltage and the conductance of the junction. An example of a conductance histogram recorded at *T* = 77 K is shown in Figure 3. Within the statistical accuracy they are in agreement with earlier findings obtained on Au contacts and realized with different methods (e.g., [18]).

**Figure 4:**
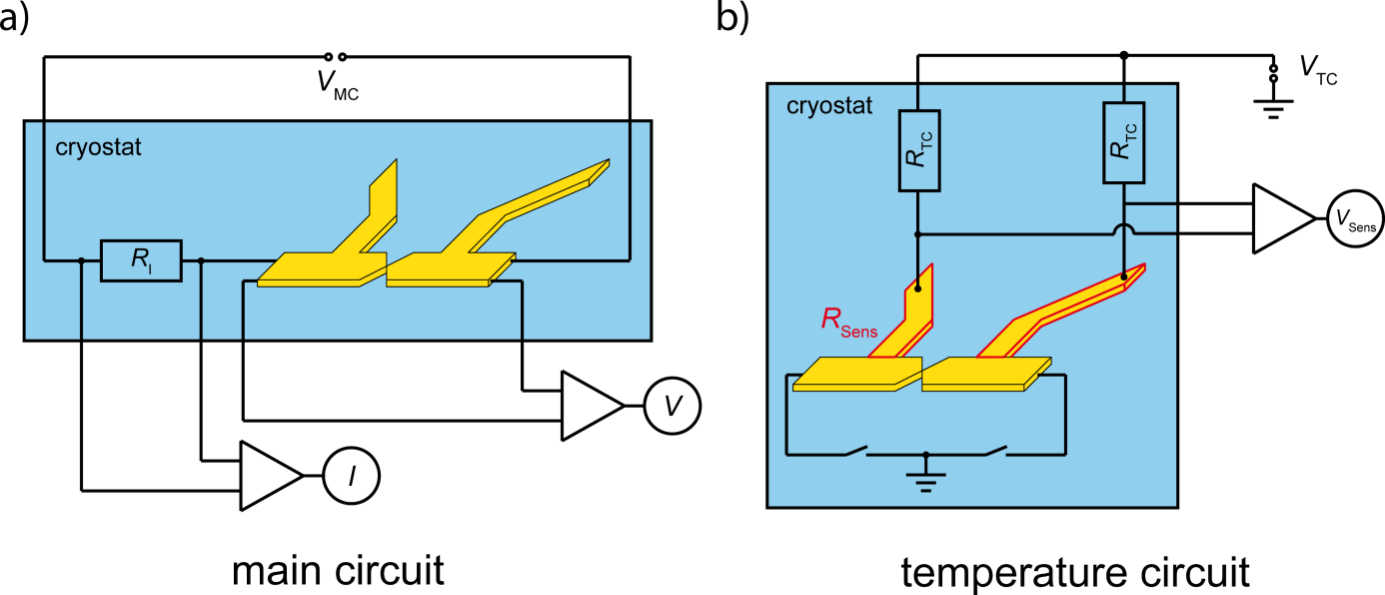
A schematic sketch of the measurement circuits. a) The main circuit where the current *I* is measured by the voltage drop across the pre-resistor *R*_I_ = 812 Ω. b) The temperature circuit with *R*_TC_ ≈ 5200 Ω and the sample connected to ground electrical potential.

For the measurement of Δ*V* and Δ*T* the standard MCBJ sample layout was modified, so that it contained two circuits: a main circuit for thermo-voltage and conductance (*G*) measurements, and a temperature circuit for the determination of the temperature change across the sample, see Figure 4.

### Determination of conductance and thermo-voltage – main circuit measurements

In the main circuit, a DC voltage (*V*_MC_) is applied and the voltage (*V*) across the junction is measured, using two different voltage amplifiers (×100 and ×20000, respectively). The amplifier with gain 100 has a very low and temporally stable voltage offset. It is used to detect the total voltage drop across the sample to determine the conductance. The high-gain amplifier serves for measuring the thermo-voltage. The current (*I*) is measured by the voltage drop across a series resistor (*R*_I_ = 812 Ω). This resistor acts as current limiter in case of a closed junction, i.e., when 1/*G* << *R*_I_. When the junction forms an atomic contact with ≈ *G*_0_, i.e., *R* >> *R*_I_, the current is dominated by the resistance of the junction.

**Figure 5:**
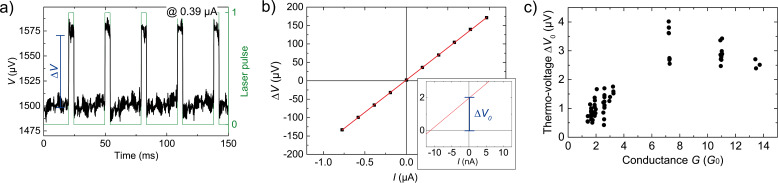
Measurement methodology of the main circuit for Au atomic-contacts at 77 K. a) Raw data of *V* versus time, at *I* = −0.39 µA, during laser pulsing (green line). ∆*V* is then be determined and averaged over many pulses at a specific *I* to achieve the ∆*V*(*I*) curve plotted in b). The fitting (red line) with ∆*V* = −175.6 Ω · *I* + 2.07 µV yields ∆*V*_0_ = ∆*V*(*I* = 0) = (2.0 ± 0.3) µV (see blow-up).The error bars determined by statistical averaging over repeated pulses are smaller than the symbol size. c) Thermo-voltage ∆*V*_0_ versus the conductance of individual contacts. For these measurements, a 514 nm laser at 1.5 mW was used to illuminate the sample at the position shown in Figure 2.

The measurement procedure for the simultaneous determination of the conductance G and the thermo-voltage Δ*V* is the following: After an atomic contact is formed, the motor is stopped. Then the measurement of *V* is performed, while the laser is pulsing on the sample with a pulse duration of 4 ms at a certain position (e.g., the one shown in Figure 2). For low voltages the current–voltage characteristic (*I*–*V*) is linear, such that *G* = *I*/*V* can be determined from the individual *V* and *I* data. The measurement resolution is in the order of 10^−2^*G*_0_. Figure 5a shows the voltage during pulsed laser heating of the substrate at the position indicated in Figure 2. The thermo-voltage (Δ*V*) is determined by the *Y*-intercept of the Δ*V*(*I*) curve with irradiation, see Figure 5c. This method ensures that all of voltage at *I* = 0 would be solely due to the thermal gradient, caused by the laser pulse [10]. To determine the thermopower of the contact the contribution of the electrical circuit to the thermo-voltage has to be determined. Taking into account the bulk value *S*_Au_ and the fact that in the present experiment the applied temperature gradient is concentrated within the Au leads on chip, this contribution is negligible [10].

The electronics are carefully calibrated before measurements are carried out to ensure that there are no other sources of bias across the junction arising from offsets of the amplifiers. Each measurement of ∆*V* is taken for a certain stable *G* value and is then repeated for contacts with different conductance. The specific measurement shown in Figure 5a,b was performed over a time interval of 50 s. A collection of ∆*V* of a number of contacts with different *G* are presented in Figure 5c.

### Determination of ∆*T* – temperature circuit measurements

**Figure 6:**
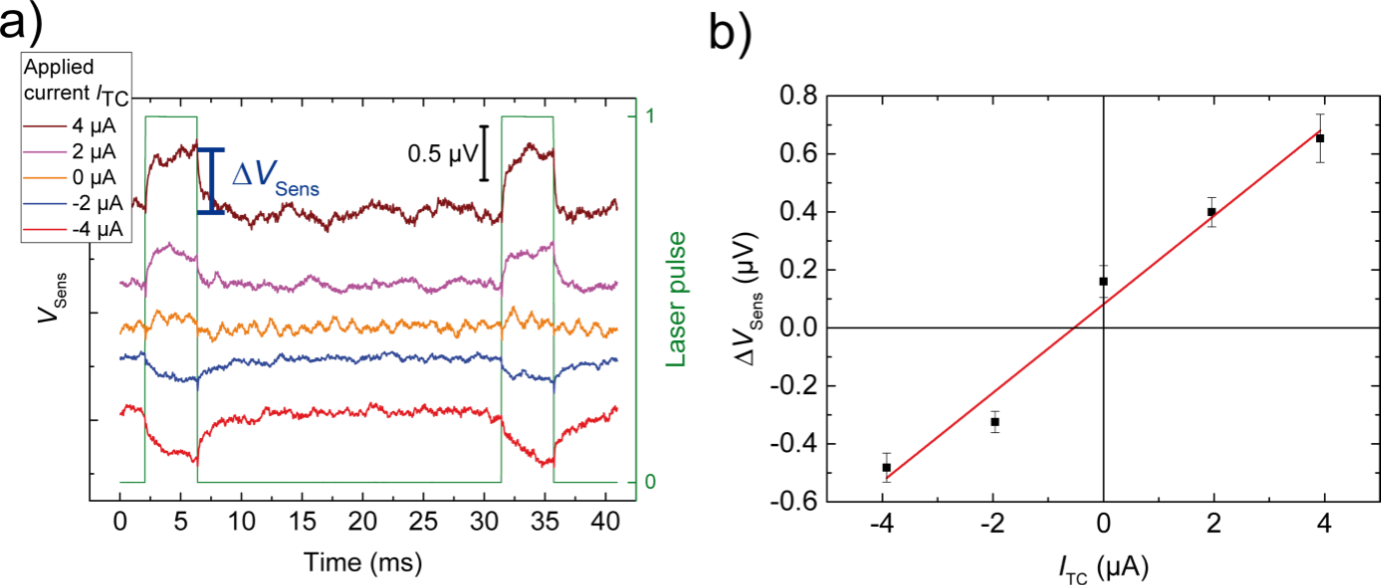
a) Raw data of *V*_Sens_ versus time at different applied currents, while the laser is pulsing on the substrate. The pulse duration of 4 ms is indicated by the green line. The curves are offset vertically to avoid overlap. b) ∆*V*_Sens_(*I*) = *V*_Sens_(on) − *V*_Sens_(off) curve, derived from the raw data in a). The error bars denote the standard deviation of the time traces while the laser was switched off, multiplied by √2 to account for the fact that the difference between on and off time is calculated. The equation of the linear fit (red line) is ∆*V*_Sens_ = 0.15 Ω · *I*_TC_ + ∆*V*_0_.

Here we suggest a method for the determination of the temperature difference across the junction for the calculation of *S*. To this end two additional Au leads have been fabricated by e-beam lithography, in one lithography step with the main circuit. The two sensor leads are located at either side of the junction, the distance between the two is about 5 µm. They have a tapered shape with a width increasing from 4 µm at the intersection with the voltage probes to 7 µm at a distance of about 100 µm from this point. In addition, they feature a kink 25 µm away from the intersection, see Figure 2. The resistance of each sensor lead (*R*_Sens_) can be measured, using a 4-point measurement scheme. Relay switches are located close to the sample to connect both junction electrodes to ground, see Figure 4b. A measurement of the voltage change across the two sensor leads (*V*_Sens_) was performed while the laser was pulsing, at different applied voltages (*V*_TC_) at the position shown in the micrograph of the sample (Figure 2) by the green dot. Figure 6a shows plots of *V*_Sens_ upon illumination for a sequence of bias currents, offset vertically for clarity. The values of ∆*V*_Sens_ are plotted versus the current *I*_TC_ in Figure 6b. A linear fit of these points results in the equation ∆*V*_Sens_ = 0.15 Ω · *I*_TC_ + 0.08 µV.

The data show two contributions of the laser heating to the observed signal, a voltage resulting from the change in the sensor lead resistance due to the temperature increase and a thermo-voltage ∆*V*_0_, which is not further considered here. These effects can be easily separated by using the relation ∆*V*_Sens_ = ∆*R*_Sens_ · *I*_TC_ + ∆*V*_0_.

The value of ∆*V*_Sens_(*I*) = *V*_Sens_(on) − *V*_Sens_(off) is defined as the change in *V*_Sens_ when the laser is pulsing (Figure 6a). The slope of the ∆*V*_Sens_(*I*_TC_) curve corresponds to the resistance change ∆*R*_Sens_, here ∆*R*_Sens_ = 0.15 Ω, which is converted into a temperature difference Δ*T* using simulations described in the next section.

### Simulations

**Figure 7:**
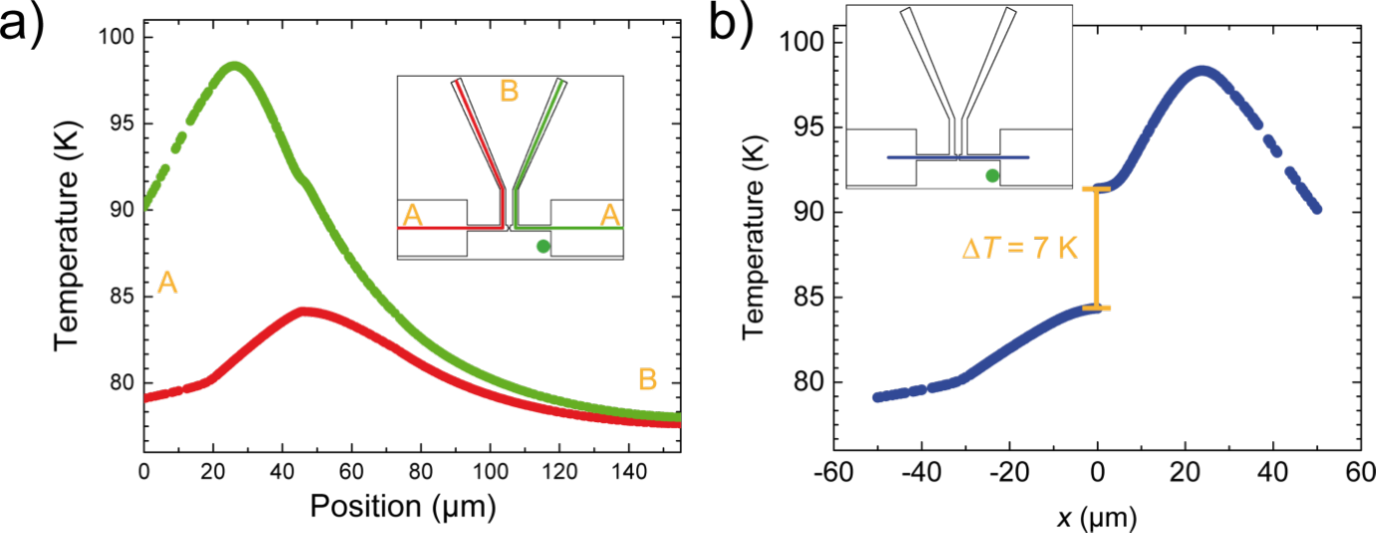
Simulations of the temperature distribution generated by a Gaussian heat source of 1.5 mW and with a diameter of 12 µm (FWHM) after 4 ms heating at a base temperature of 77 K. We implemented a structure similar to our device. The irradiation point (green dot) corresponds to the one used in our experiment. We calculate the temperature profile along different cross sections shown in the insets as red, green (a) and blue lines (b) (see insets). a) The temperature behavior along the red trace (red curve) and the green trace (green curve). b) The temperature behavior along *y* = 0 µm where the junction is located at position (0,0). The simulation shows a temperature difference of about 7 K.

In order to derive ∆*T* at the junction from the resistance difference ∆*R*_Sens_ measured between the two sensor leads, finite elements simulations were carried out using COMSOL Multiphysics [26]. Simulations were performed for the equivalent sample design, with 63 × 100 nm^2^ cross section of the suspended Au bridge. To simulate the fact that the measurement was performed at the relatively low conductance of 4*G*_0_, we assume that the contact is thermally and electrically insulating. The gold structure was placed on a Kapton Cirlex substrate (properties from COMSOL Material Library: Polyimide tape (Kapton HN)) with an additional polyimide layer (properties from COMSOL MEMS module: Polyimide) of 2.5 µm thickness. A Gaussian heat source with 1.5 mW and a diameter of 12 µm (FWHM) was located on the substrate surface to represent a λ ≈ 500 nm laser source [25]. Simulations were carried out for 77 K at the position of the laser spot aside of the metal film as in the experiment (Figure 7 insets and Figure 2).

We investigated the temperature profile along three cross sections after 4 ms of heating. Two of them are shown in Figure 7a (green and red curves and traces), Figure 7b illustrates the third cross section at *y* = 0, wherein the junction is located at (0,0). ∆*T* was calculated as the difference in temperature between both sides of the constriction, see Figure 7b, here ∆*T* = 7 K.

**Figure 8:**
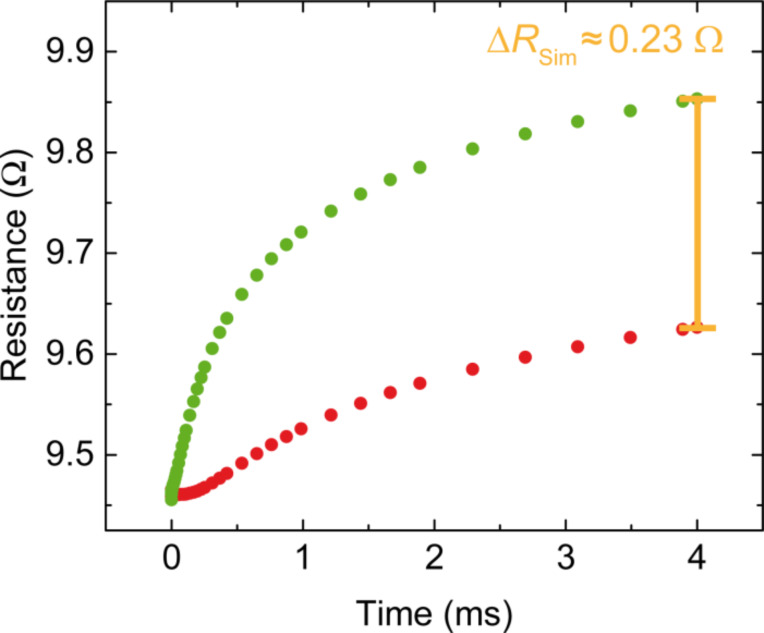
Time-dependent simulation of the resistivity of the two sensor leads due to heating. After 4 ms the resistance change amounts to ∆*R*_Sim_ = 0.23 Ω.

To determine the resistance change caused by this temperature difference we also simulate the time-dependent sensor leads resistance due to laser heating. The simulation was fed with measured resistance values of a sample which had the same dimensions and fabrication parameters as the one used in the experiment. As usual for thin films the resistivity is higher than estimated from bulk data [27]. The nominal experimental thickness of the Au films was 80 nm, but by comparison with literature data obtained on thin gold films on different substrates [28] and assuming that at temperatures above 200 K the temperature dependence of the resistivity should be dominated by electron-phonon scattering, we obtained an effective thickness of 63 nm. This reduction can be caused by the formation of a dead layer or by increased roughness due to the etching process. The complete length of the sensor leads including the parts connecting to ground, (see Figure 7a, red and green line) was taken into account. The simulation results are shown in Figure 8. Again, the green curve corresponds to the right sensor lead and the red curve to the left one. After 4 ms of heating the resistance difference of the two sensor leads amounts to ∆*R*_Sim_ = 0.23 Ω, in the same range as the experimental finding of 0.15 Ω.

The 50% larger resistance difference is attributed to the assumption of completely suppressed heat transfer across the junction, leading to an overestimation of the temperature and resistance differences. Nevertheless the comparison between experiment and simulation validates the experimental approach and enables the calibration of the temperature difference across the junction (∆*T*).

**Figure 9:**
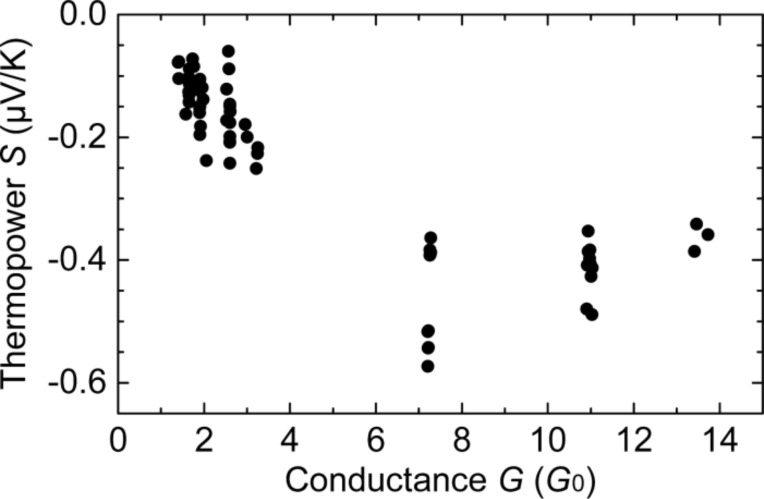
Thermopower *S*(*G*) versus conductance *G* of individual contacts, calculated from the ∆*V*(*G*) data shown in Figure 5d and the corresponding ∆*T* (=7 K) measurement shown in Figure 7b.

With the experimentally determined values for ∆*V* and ∆*T*, *S* can also be derived. Figure 9 shows an example for *S*(*G*) data, calculated from ∆*V*(*G*), presented in Figure 5c with the corresponding ∆*T*, presented in Figure 7b. The sign of *S* is negative and its values are in good agreement with previous studies of Au atomic contacts [10,12]. While in [12] pronounced fluctuations with an average value of *S* = 0 was observed, in [10] *S* was shown to adopt both signs as well, but the mean value is negative for atomic contacts with *G* up to 10^3^*G*_0_.

Our data confirms the negative sign. However, the variance is smaller such that all measured *S* values are negative, probably because of the smaller statistical ensemble. A more comprehensive study of the thermopower of single-atom contacts will be the topic of a forthcoming publication.

## Conclusion

To conclude, we have shown a new method for a measurement of the temperature difference across an atomic-scale device which will increase future applicability of thermo-voltage measurements. Our setup enables simultaneous measurements of the conductance and the thermo-voltage at low temperature. At variance to other realizations the temperature gradient is created here contact-free by irradiation with laser light, and the detection of the temperature and the voltage difference is resolved in time. The possibility to vary the position of the heating allows in principle to investigate geometry-dependent effects. Another novel ingredient for thermopower measurements is the combination of finite element simulations and the usage of the temperature dependence of the resistive leads for estimating the temperature gradient. This combination allows the determination of the Seebeck coefficient of atomic-scale devices, which will lead to a better understanding of charge transport in these systems. The set-up is also suitable for measuring the thermopower of single-molecule contacts as well as to be operated at variable base temperature. While the signal size of the thermopower scales with the temperature, the stability of the junctions and the purity of the contacts due to the higher vacuum are improved at low temperature and therefore enable more detailed studies of the thermopower for instance as a function of bias voltage. The cryogenic vacuum at low temperatures makes it possible to study atomic-contacts of more reactive metals commonly used in quantum transport studies or in molecular electronics like silver, platinum, aluminum or others. In particular, the study of single-molecule junctions, which at room temperature suffer from reduced lifetime, is largely facilitated. Furthermore, temperature-dependent effects of the transmission function that are expected in resonant tunneling situations can be revealed. Thus the ability to measure at variable temperature represents a considerable improvement compared to fixed-temperature set-ups.

## Methods

### Estimation of the laser spot size

**Figure 10:**
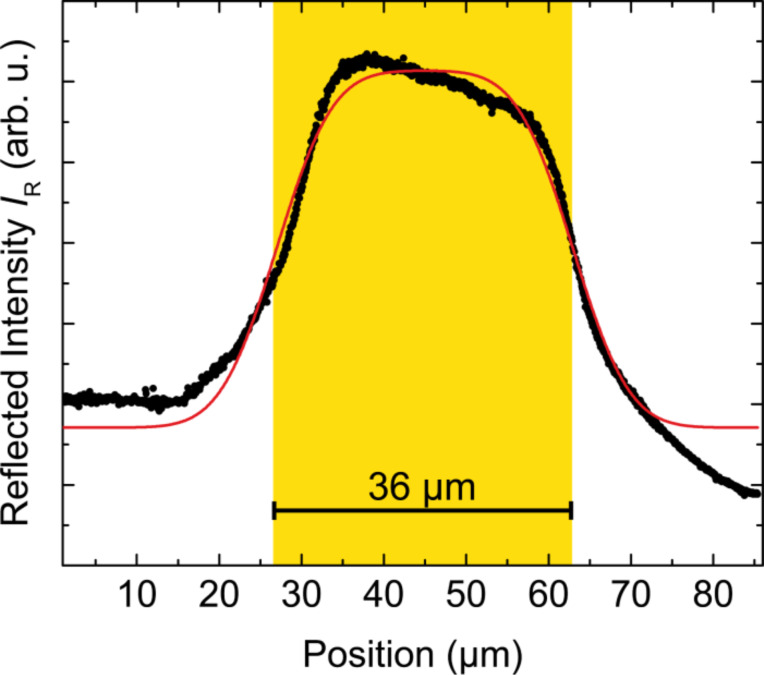
Reflected intensity versus the position of the laser spot across the 36 µm wide gold stripe. The spot size was determined by the knife-edge technique to 12.7 µm. The red curve is the fit with *I*_R_ = *A* · (erf(*x* – *b*_0_ + *b*/2)/*s*) – (erf(*x* – *b*_0_ − *b*/2)/*s*) + *c*, where *b* = 36 µm and *s* = 7.6 µm.

To estimate the diameter of the laser spot we used the knife edge technique. Thereby the laser spot is moved across a 36 µm wide (determined by optical microscope) stripe of gold while the reflected intensity is detected with a photodiode. Due to the different reflectivity of gold and Kapton Cirlex there is an intensity profile corresponding to the convolution of a box function for the stripe and a 2D Gauss profile for the laser. It can be easily shown that the direction parallel to the stripe does not play any role, so the convolution reduces to a 1D integral, which leads to a difference of two error functions, which is used to fit the dataset. Figure 10 shows the reflected intensity *I*_R_ with the fit *I*_R_ = *A* · [erf((*x* – *b*_0_ + *b*/2)/*s*) – ((erf(*x* – *b*_0_ − *b*/2)/*s*)] + *c*, where *b* = 36 µm and *s* = 7.6 µm. The formula to calculate the spot size leads to 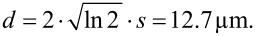 All other parameters, *A*, *b*_0_, and *c* are not relevant for the determination.

### Sample details

**Figure 11:**
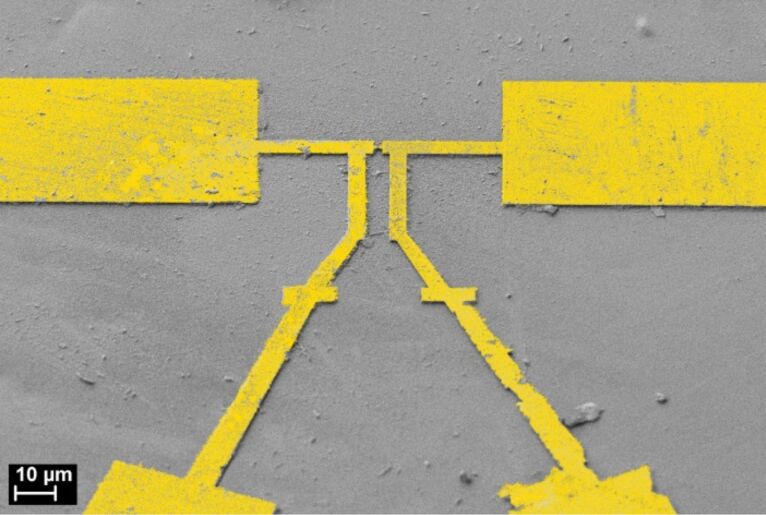
Colored scanning electron micrograph of the sample shown in Figure 2, recorded after the measurements under an angle of 45°.

### Simulation details

For the simulations we used the following material parameters: Gold: Thermal conductivity κ = 320/1.85 W m^−1^ K^−1^, specific heat *c*_p_ = 128 J kg^−1^ K^−1^, mass density ρ_m_ = 19300 kg m^−3^, electrical resistivity ρ(*T*) = 8.73575 × 10^−9^ Ωm + 0.08325 × 10^−9^ Ωm K^−1^·*T*; polyimide: κ = 0.15 W m^−1^ K^−1^, *c*_p_ = 1100 J kg^−1^ K^−1^, ρ_m_ = 1300 kg m^−3^; Kapton: κ = (−1.372384 × 10^−3^ + 5.601653 × 10^−3^·*T* + 2.082966 × 10^−6^·*T*^2^ − 5.05445 × 10^−9^·*T^3^*) W m^−1^ K^−1^ (for 5 K ≤ *T* < 140 K), κ = (−7.707532 × 10^−3^ + 5.769136 × 10^−3^·*T* + 5.622796 × 10^−7^·*T*^2^ − 4.329984 × 10^−10^·*T*^3^) W m^−1^ K^−1^ (for 140 K ≤ *T* ≤ 300 K) [29–30]; *c*_p_ = (2.809666 − 1.394349·*T*^−1^ + 0.2106639·*T*^−2^ + 4.752016 × 10^−3^·*T*^−3^ − 3.279998 × 10^−4^·*T*^−4^ + 4.282249 × 10^−6^·*T*^−5^) J kg^−1^ K^−1^ (for 4 K ≤ *T* < 30 K), *c*_p_ = −86.86946 + 7.816917·*T* − 0.03664788 × 10^−2^·*T*^−2^ + 9.9128 × 10^−5^·*T*^−3^ − 1.08441 × 10^−7^·*T*^−4^ J kg^−1^ K^−1^ (for 30 K ≤ *T* ≤ 300 K) [30], ρ_m_ = 1420 kg m^−3^.
